# Enzymatic synthesis using glycoside phosphorylases

**DOI:** 10.1016/j.carres.2014.06.010

**Published:** 2015-02-11

**Authors:** Ellis C. O’Neill, Robert A. Field

**Affiliations:** Department of Biological Chemistry, John Innes Centre, Norwich Research Park, Norwich NR4 7UH, UK

**Keywords:** Phosphorylases, Glycosides, Synthesis

## Abstract

Carbohydrate phosphorylases are readily accessible but under-explored catalysts for glycoside synthesis. Their use of accessible and relatively stable sugar phosphates as donor substrates underlies their potential. A wide range of these enzymes has been reported of late, displaying a range of preferences for sugar donors, acceptors and glycosidic linkages. This has allowed this class of enzymes to be used in the synthesis of diverse carbohydrate structures, including at the industrial scale. As more phosphorylase enzymes are discovered, access to further difficult to synthesise glycosides will be enabled. Herein we review reported phosphorylase enzymes and the glycoside products that they have been used to synthesise.

## Highlights

• An extensive survey of the currently known carbohydrate phosphorylases is presented.
• The utility of phosphorylases in glycoside synthesis is discussed.
• Future directions for the discovery and development of phosphorylases for use in synthesis are proposed.

## Graphical abstract

**Figure:**
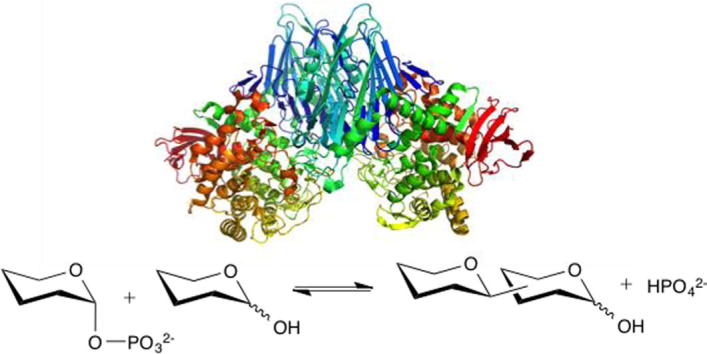

## 1 Introduction

**Figure 1:**
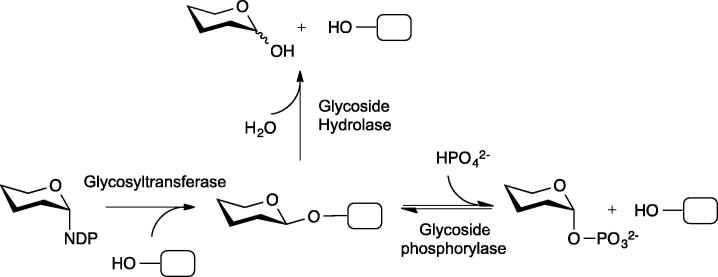
Reactions performed by glycosyltransferases, glycoside hydrolases and glycoside phosphorylases. NDP = nucleotide diphosphate.

Enzymatic synthesis of glycans represents an attractive alternative to chemical synthesis, avoiding the need for tedious and extensive protecting group chemistry. Much effort has focused on the exploitation of naturally catabolic glycoside hydrolases, and mutants thereof, in synthesis; glycosyltransferases have also received much attention, in spite of occasional issues with enzyme stability and accessibility of sugar nucleotide donor substrates.^1,2^ In contrast, glycoside phosphorylases have received much less attention for the biotransformation of carbohydrates until recently. These latter enzymes utilise accessible and relatively stable sugar-1-phosphates in effecting stereo- and regio-selective synthesis of glycosidic linkages (Fig. 1).^3^ This has allowed their use in the synthesis of diverse carbohydrate structures, including the commercial synthesis of 2-*O*-α-d-glucosyl glycerol (**31**)^4^ and the kilogram scale synthesis of lacto-*N*-biose (**56**)^5^ (vide infra).

**Figure 2:**
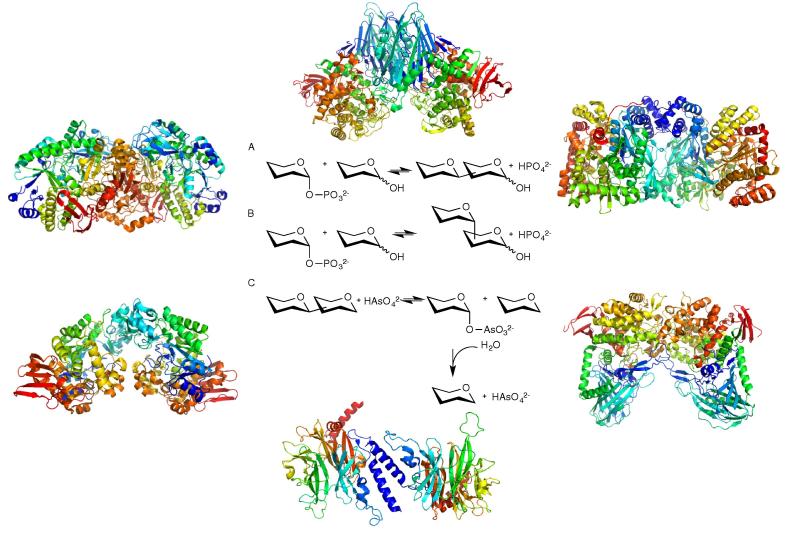
General scheme for phosphorylase actions and representatives of the six families for which structures have been solved. (A) The reaction of inverting phosphorylases. (B) The reaction of retaining phosphorylases. (C) Arsenolysis. GH112, GH94, GT35, GH65, GH130 and GH13, clockwise from top left. (2ZUS,^61^ 3QDE,^141^1GPA,^65^ 1H54,^142^ 4KMI^127^ and 1R7A^137^). Replacement of inorganic phosphate with arsenate releases an unstable sugar-arsenate, which readily decomposes to the free monosaccharide, giving rise to net glycoside hydrolysis.^143^

The currently characterised glycoside phosphorylases are soluble enzymes that catalyse the reversible addition of phosphate across glycosidic linkages in a stereospecific manner (Fig. 2).^6^ The phosphorylase reaction may be inverting or retaining with respect to the glycosidic linkage formed, which is determined by their reaction mechanism and is reflected in their classification in CAZy families.^7^

A recent article from Nakai et al. provides a useful overview of phosphorylase structure and mechanism.^3^ Herein we review the utilisation of phosphorylases in glycoside synthesis, providing an update on earlier reviews of this topic.^3,6,8^ In a number of instances, the kinetic characterisation of phosphorylase action has been reported but the products of such experiments have not been fully characterised. Nonetheless, such examples are included herein in order to project the potential of phosphorylases as synthesis tools.

**Figure 3:**
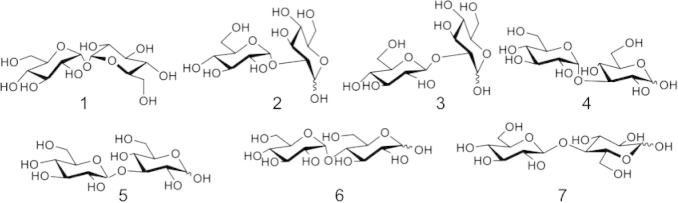
d-Glc-d-Glc disaccharide linkages for which phosphorylases are found in Nature. Trehalose (α,α-1,1-d-glucosyl d-glucose) (**1**). Kojibiose (α-1,2-d-glucosyl d-glucose) (**2**). Sophorose (β-1,2-d-glucosyl d-glucose) (**3**). Nigerose (α-1,3-d-glucosyl d-glucose) (**4**). Laminaribiose (β-1,3-d-glucosyl d-glucose) (**5**). Maltose (α-1,4-d-glucosyl d-glucose) (**6**). Cellobiose (β-1,4-d-glucosyl d-glucose) (**7**).

Phosphorylases have been found for many types of glycosides, but most identified to date act on d-glucosyl residues (Table 1). In fact, there are characterised disaccharide phosphorylases for every conceivable d-Glc-d-Glc linkage except β-1,1 and α- or β-1,6 (Fig. 3).

In the following sections, current state of knowledge of the various classes of glycoside phosphorylases is outlined.

## 2 α,α-1,1-d-Glucan phosphorylases

**Figure 4:**
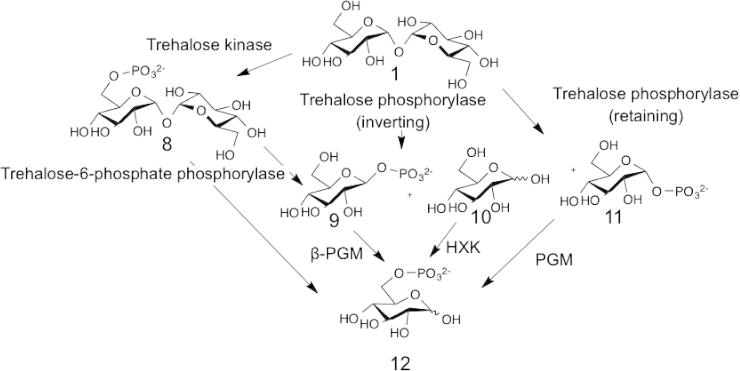
Trehalose metabolism using phosphorylases. (A) Trehalose (**1**) can be phosphorylated to trehalose-6-phosphate (**8**) by trehalose kinase before being phosphorolysed by an inverting phosphorylase yielding Glc-6-P (**12**) and β-Glc-1-P (**9**), which is converted into Glc-6-P (**12**) by β-phosphoglucomutase (β-PGM).^17^ (B) Trehalose (**1**) is phosphorolysed by inverting phosphorylases from GH65 to form β-Glc-1-P (**9**) and Glc (**10**) which are converted into Glc-6-P (**12**) by β-phosphoglucomutase and hexokinase (HXK), respectively.^13^ (C) Retaining trehalose phosphorylase from family GT4 can convert trehalose (**1**) to α-Glc-1-P (**11**) and Glc (**10**), which are converted into Glc-6-P (**12**) by phosphoglucomutase (PGM) and hexokinase, respectively.^20^

Trehalose (d-Glc-α,α-1,1-d-Glc, **1**), which can accumulate to very high levels in a variety of bacteria, fungi, insects and plants,^9^ is used in many different biological roles, including in energy storage,^10^ in abiotic stress responses^11^ and for osmoregulation.^12^ Trehalose metabolism can be by hydrolysis, but it is often by phosphorolysis (Fig. 4).

### 2.1 Inverting trehalose phosphorylase

**Figure 5:**
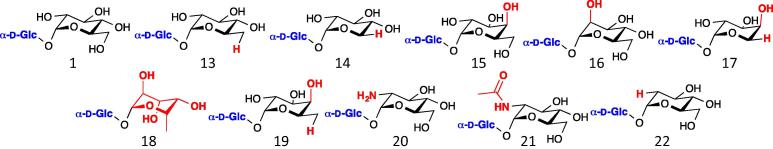
Proposed products formed using trehalose phosphorylase. (A) *Euglena* trehalose phosphorylase was able to make glycosides (product number in brackets) by transfer of d-glucose onto d-glucose (forming **1**), 6-deoxy-d-glucose (**13**) and d-xylose (**14**).^14^ (B) *Thermoanaerobium* trehalose phosphorylase was able to transfer glucose onto d-galactose (**15**), d-mannose (**16**), l-arabinose (**17**), l- (**18**) and d- (**19**) fucose, d-glucosamine (**20**), *N*-acetyl-d-glucosamine (**21**) and 2-deoxy-d-glucose (**22**).^16^ These latter experiments only assess Pi release from d-Glc-1-P by the phosphorylase in the presence of these acceptors; product structures have not been confirmed.

The protozoan alga *Euglena gracilis* produces an enzyme that shows phosphorolytic activity towards trehalose.^13^ In the glycoside synthesis sense, this enzyme activity shows promiscuity towards the 6 position of the acceptor, with both 6-deoxy-d-glucose and d-xylose serving as acceptor substrates, giving glucosides (**13**) and (**14**), respectively (Fig. 5).^14^ A similar activity has also been identified in bacteria, including plant symbionts^15^ and thermophiles.^16^ In the latter case, this activity was shown to be promiscuous with respect to acceptor configuration, as highlighted in Figure 5.^16^

### 2.2 Trehalose-6-phosphate phosphorylase

As part of their normal trehalose metabolism, many acid bacteria phosphorylate the trehalose and then use trehalose-6-phosphate phosphorylase^17^ to release β-d-Glc-1-P (**9**) and d-Glc-6-P (**12**) into the hexose phosphate pool (Fig. 4).^18^

### 2.3 Retaining trehalose phosphorylase

An alternative trehalose phosphorylase was identified in *Flammulina velutipes* which is retaining, and thus produces α-d-Glc-1-P (Fig. 4, **11**).^19^ This enzyme has since been found in many other fungi,^20^ but it has not yet been the subject of investigation for use in synthesis.

## 3 α-1,2-d-Glucan phosphorylases

### 3.1 Kojibiose phosphorylase

In nature kojibiose, (d-Glc-α-1,2-d-Glc, **2**), is found in honey^21^ and as a component of *Leuconostoc* dextran,^22^ for instance, whilst Kojidextran is produced by *Rhizobium* species.^23^ It is also part of the core antigen of *Moraxella catarrhalis*, which is currently under investigation as a vaccine candidate for the prevention of human respiratory tract infections.^24^

**Figure 6:**
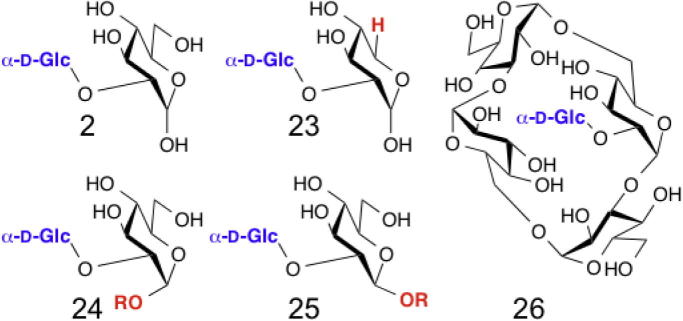
Prospective products formed using kojibiose phosphorylase. Glucose can be transferred (product number in brackets) onto glucose (**2**), d-xylose (**23**) and α (**24**) and β (**25**) d-glucosides (R = Me or Glc).^25^ A cyclic tetrasaccharide was glycosylated with this enzyme (**26**).^29^

A phosphorylase isolated from *Thermoanaerobium brockii* was shown to be capable of degrading kojibiose (**2**) to yield β-Glc-1-P.^25^ This kojibiose phosphorylase was also capable of the reverse reaction, consecutively transferring glucose residues to make kojioligosaccharides (up to d.p. 7),^26^ and the enzyme from *Caldicellulosiruptor saccharolyticus* can be used for the synthesis of longer oligosaccharides (with a larger proportion of molecules with d.p. >3).^27^ Mutagenesis or chimera generation with the kojibiose phosphorylase and the trehalose phosphorylase from the same organism allowed even longer kojioligosaccharide to be formed (up to d.p. 14).^28^ The enzyme transferred d-glucose onto d-*gluco*-configured sugars, but tolerated either anomeric configuration and a range of anomeric substituents in the d-glucoside acceptor (Fig. 6).^25^

Beyond simpler sugar acceptors, this enzyme could be used for the addition of d-glucose to a cyclic α-d-glucan, forming *cyclo*-{d-Glc-α-1,3-d-Glc-[α-1,2-d-Glc-]-α-1,6-d-Glc-α-1,3-d-Glc*p*-α-1,6-} (**26**).^29^ This enzyme was also noted as transferring d-glucose onto l-sorbose and l-xylose, though it is unclear to which hydroxyl group the glycosidic bond is formed.^25^

### 3.2 Sucrose phosphorylase

**Figure 7:**
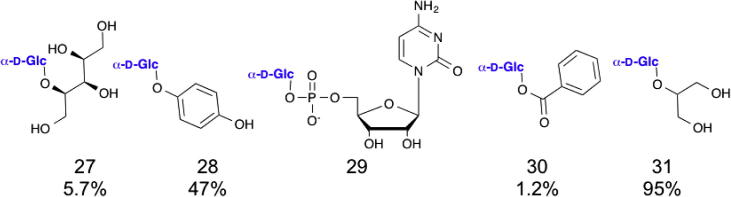
Examples of products made with sucrose phosphorylases. Sucrose phosphorylase has been used to transfer glucose (product number in brackets) onto xylitol (**27**),^32^ hydroquinone (**28**),^33^ cytosine mono phosphate (**29**),^36^ benzoic acid (**30**)^35^ and glycerol (**31**) (isolated yields noted where reported).^4^

Sucrose phosphorylase is found in diverse bacteria, such as *Leuconostoc mesenteroides*,^30^ where it catalyses the phosphorolysis of sucrose (d-Glc-α,α-1,2-d-Fru), releasing α-d-Glc-1-P and d-fructose.^31^ This enzyme is a member of the GH13 family and its very broad acceptor specificity has led to it being widely used for the in vitro transfer of d-glucose, including onto sugars, sugar alcohols^32^ and phenols.^33^ A range of sucrose phosphorylases from different bacteria have been explored for their acceptor specificity^34^ and transfer onto carboxylic acids^35^ and even the phosphate group of a nucleotide^36^ has been achieved (Fig. 7). For a review of sucrose phosphorylase-catalysed *trans*-glucosylation, readers are referred to Goedl et al.^37^ The flexibility and robustness of this class of enzyme has enabled the large scale synthesis of a variety of compounds, including the gram scale synthesis of α-d-glucosyl d-xyitol (**27**)^32^ and α-arbutin (*O*-α-d--glucosyl hydroquinone, **26**).^33^ This enzyme is now used for the industrial scale synthesis of 2-*O*-α-d-glucosyl glycerol (**31**).^4^

### 3.3 d-Glucosyl glycerol phosphorylase

Recently an enzyme was identified in *Bacillus selenitireducens* that belongs to the family GH65 inverting phosphorylases.^38^ A high rate of hydrolysis of β-d-Glc-1-P was measured with this enzyme, with no glycoside formation in the presence of prospective monosaccharide acceptors. However, in the presence of glycerol a new product was formed that was identified as 2-*O*-α-d-glucosyl glycerol (**31**), in an overall yield of 47% on a 300 mg scale.^38^

## 4 β-1,2-d-Glucan phosphorylases

Some bacteria, including *Rhizobia*^39^ and *Brucella*,^40^ synthesise cyclic β-1,2-glucans as osmoregulators, which are necessary for virulence.^41^ The synthesis of these compounds proceeds at the cell membrane by initial auto-glucosylation of the enzyme,^42^ extension of the growing chain and cyclisation once the chain reaches the appropriate length. This length control is mediated by the C-terminal domain of the protein, which is an inverting GH94 family phosphorylase.^43^ To date, this enzyme has not been explored for the synthesis of oligosaccharides.

## 5 α-1,3-d-Glucan phosphorylases

### 5.1 Nigerose phosphorylase

**Figure 8:**
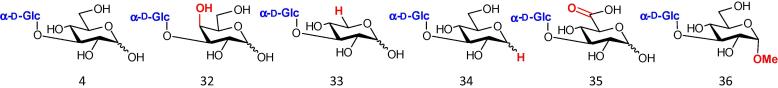
Prospective products formed using nigerose phosphorylase. Glucose can be transferred onto (product number in brackets) d-glucose (**4**), d-galactose (**32**), d-xylose (**33**), 1,5-anydro-d-glucitol (**34**), d-glucuronic acid (**35**) and methyl α-d-glucoside (**36**).^44^ Only kinetic parameters were reported and no products were isolated.

Recently an enzyme that degrades α-1,3-d-glucosyl d-glucose (nigerose, **4**) was identified in *Clostridium phytofermentans* and has been characterised as nigerose phosphorylase.^44^ α-1,3-Glucan polymers are found in fungal cell walls^45^ and this is presumably the source of the natural substrate for this enzyme. The enzyme shows some activity towards kojibiose (**2**) as an acceptor and can also transfer a single glucose residue onto some other monosaccharides (Fig. 8).

### 5.2 d-Glucosyl l-rhamnose phosphorylase

An α-1,3-d-glucosyl l-rhamnose phosphorylase has been identified in *Clostridium phytofermentans*.^46^ The sugar substrate for this enzyme is found as a component of plant and bacterial cell walls; in this instance, it has been postulated that the enzyme is involved in Clostridial cell wall recycling. In an in vitro experiment, this phosphorylase was capable of synthesising α-1,3-glucosyl l-rhamnose disaccharide in 64% yield on a 5 mg scale; no other sugars tested were acceptors for this enzyme.^46^

## 6 β-1,3-Glycan phosphorylases

### 6.1 β-1,3-d-Glucan phosphorylases

β-1,3-Glucans (e.g. laminarins) are used in some algae, including *Euglena* and brown algae, as their major storage carbohydrate^47^ and make up the cell walls of some fungi, including yeasts and Basidiomycetes.^48^ β-1,3-Glucan phosphorylases are categorised into three sub-groups based on substrate chain length preference.

**Figure 9:**
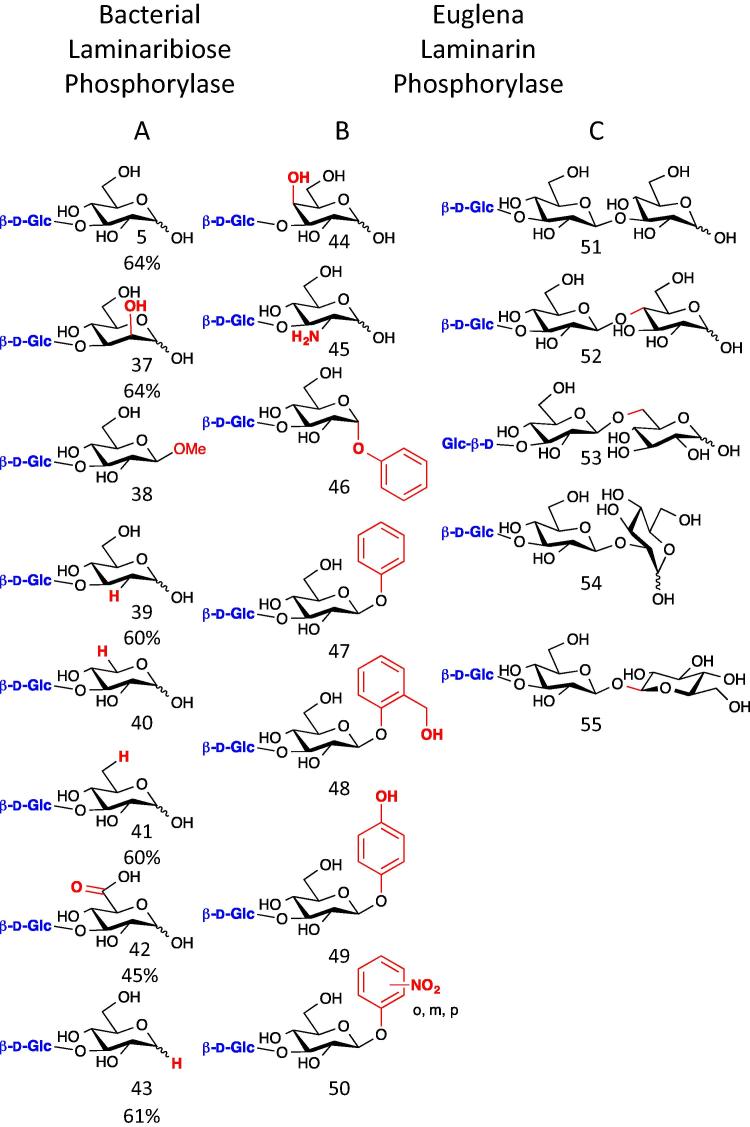
Proposed products formed using laminarin phosphorylases. (A) Proposed products (compound numbers in brackets) formed when glucose was transferred by bacterial laminaribiose phosphorylases on to Glc (**5**), Man (**37**), β-methyl d-glucoside (**38**), 2-deoxy-d-glucose (**39**), d-xylose (**40**), 6-deoxy-d-glucose (**41**), d-glucuronic acid (**42**) and 1,5-anhydroglucitol (**43**).^57^ (B) Phosphorylases from *Euglena* are capable of transferring glucose on to the same sugars as the bacterial phosphorylases and in addition d-galactose (**44**), d-glucosamine (**45**), α- (**46**) and β-phenyl (**47**) d-glucosides, salicin (**48**), arbutin (**49**)^51^ and *o*/*m*/*p*-nitrophenyl β-glucosides (**50**).^54^ (C) Glucose can also be transferred on to laminaribiose (**51**), cellobiose (**52**), gentiobiose (**53**), sophorose (**54**)^54^ and β,β-trehalose (**55**).^50^ Reasonable yields were obtained for some of the products but for many only kinetic parameters were measured.

Laminaribiose phosphorylases were first discovered in *Euglena gracilis*^49^ and related algae,^50^ whilst laminarin phosphorylase^51^ and 1,3-β-glucan phosphorylase, with preferences for longer glucans, have also been identified in algae and plants.^52,53^ The *Euglena* enzyme has been partially purified and analysed,^54^ although no sequence data are available. This enzyme has proved useful in the synthesis of plant cell wall related oligosaccharides, including mixed linkage β-glucans (**135**–**138**),^55^ and for the β-1,3-glucosylation of monosaccharides such as galactose and glucosamine, giving glucosides (**44**) and (**45**), respectively (Fig. 9).^51^

Two laminaribiose phosphorylases have been cloned from β-1,3-glucan-metabolising bacteria, *Paenibacillus* sp. *YM-1* and *Acholeoplasma laidlawii*.^56,57^ The substrate flexibility of these GH94 family enzymes has been explored and showing reasonable promiscuity towards substitution at the 2 and 6 positions of the acceptor substrate (see **37**, **39**; **40**, **41**, **42**). These enzymes are strict disaccharide phosphorylases.

### 6.2 d-Galactosyl d-HexNAc phosphorylase

**Figure 10:**
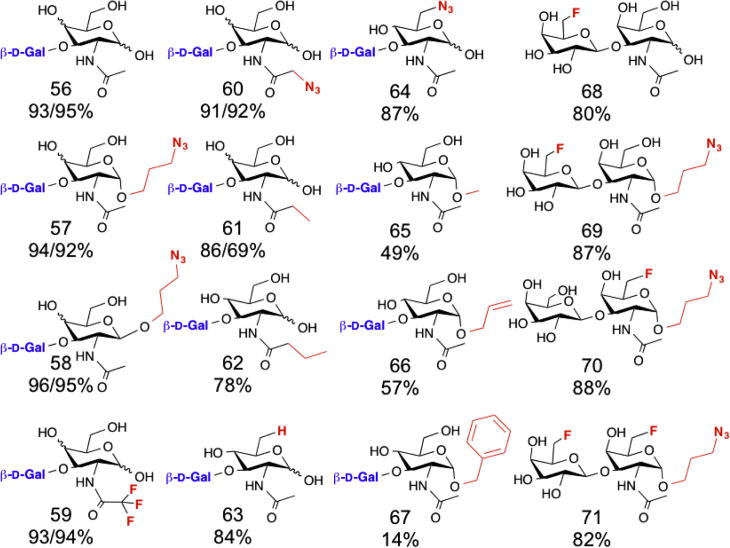
Disaccharide formed using β-1,3-GalHexNAc phosphorylase. Galactose was transferred (product numbers in brackets) onto the 3 OH of GlcNAc and GalNAc (**56**), α (**57**) and β (**58**) anomeric propyl azides, and the acetate replacements trifluoroacetate (**59**), azidoacetate (**60**) and propanoate (**61**). Only the glucose configured *N*-butanoyl (**62**), 6-deoxy (**63**), 6-azido-6-deoxy (**64**),^63,64^ α-methyl (**65**), allyl (**66**) and benzyl (**67**) glycosides^144^ were used as acceptors. 6-Deoxy-6-fluoro-d-galactose could also be transferred onto Gal (**68**), α-azidopropyl Gal (**69**) and α-azidopropyl 6-fluoro-6-deoxy Gal was an acceptor for transfer of both Gal (**70**) and 6-deoxy-6-fluoro Gal (**71**), though glucose configured acceptors were not tested.^64^ Yield for Glc/Gal configured acceptors where both were achieved.

Human mucin^58^ and milk^59^ contain complex oligosaccharides that are degraded by intestinal bacteria, such as *Bifidobacterium bifidum*, in part by hydrolysis, but also by phosphorolysis.^60^ These enzymes, which belong to the GH112 family,^61^ release d-Gal-1-P from Gal-β-1,3-GlcNAc or Gal-β-1,3-GalNAc (**56**).^62^ Using the *B. infantis* enzyme, a range of substituted and unsubstituted disaccharides was prepared by transferring d-galactose onto the 3-OH of various monosaccharide acceptors (Fig. 10).^63^ This approach enabled the synthesis of fluorinated T antigen derivatives (**68**–**71**).^64^

## 7 α-1,4-d-Glucan phosphorylases

### 7.1 α-1,4-d-Glucan phosphorylases

α-1,4-d-Glucan phosphorylases are present in all classes of organism from bacteria (maltodextrin phosphorylase) to animals (glycogen phosphorylase) and plants (starch phosphorylase). Structural studies of these mammalian,^65^ bacterial,^66^ yeast^67^ and plant^68^ retaining phosphorylases identify them as members of the glycosyltransferase family 35, which uniquely among the phosphorylases require a pyridoxal phosphate prosthetic group to act as the general acid/base for catalysis.^69^ The physiological function of this class of enzymes appears to be in the metabolism of glucan storage macromolecules, though they all have slightly different specificities for chain length and branching frequency, as expected given their different physiological substrates (glycogen in mammals and bacteria; starch in plants).

**Figure 11:**
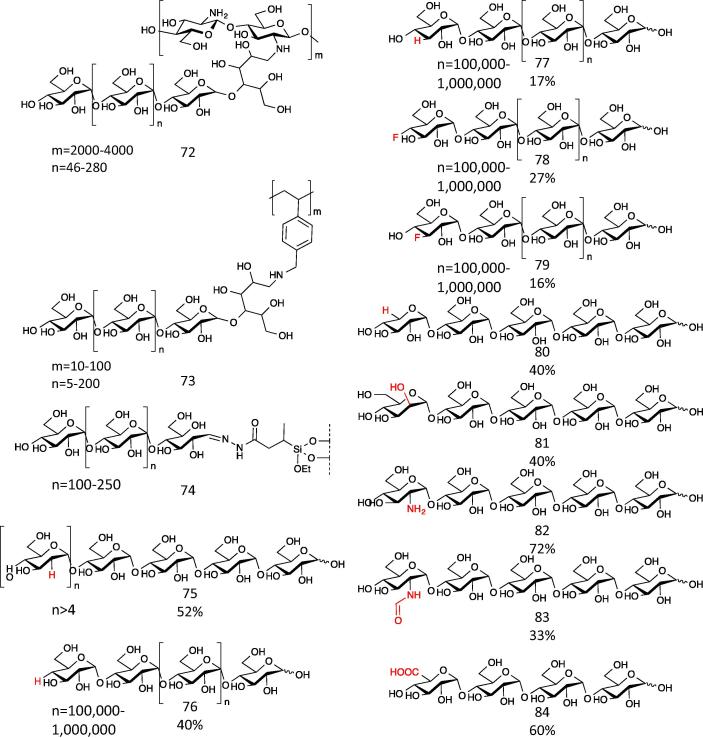
Action of α-1,4-glucan phosphorylases on non-natural acceptor and donor substrates. Phosphorylases were used to extend (product numbers in brackets) various maltooligosaccharide grafted compounds to form, for example, amylose grafted chitosan (**72**),^73^ polystyrene (**73**)^74^ and HPLC matrix (**74**).^76^ Phosphorylase could transfer various sugars from their respective anomeric phosphates on to acceptors. 2-Deoxy-α-1,4-d-glucans were synthesised from d-glucal (**75**),^78^ which can then be phosphorolysed to synthesise 2-deoxy-α-Glc-1-P,^145^ and 3- and 4-deoxy and fluoro-d-glucose were transferred on to glycogen (**76**–**79**) by rabbit muscle enzyme.^79^ Potato phosphorylase has been used to transfer d-xylose (**80**),^80^d-mannose (**81**),^81^d-glucosamine (**82**)^82^ and *N*-formyl-d-glucosamine^83^ (**83**) from their respective phosphates on to maltotetraose. A thermostable phosphorylase from *Aquifex aeolicus* catalysed the transfer of d-glucuronic acid on to maltotriose (**84**).^84^ Yields are indicated below for some of the products. For some examples, no yield was calculated but sufficient product was obtained for characterisation by NMR.

α-1,4-Glucan phosphorylases represent an amenable system for the synthesis of extended amylose chains up to 80 or more residues^68^ whilst retaining control over the chain length.^70^ Mammalian glycogen phosphorylases have proven less useful in biotechnology than plant phosphorylases because of their allosteric regulation, which is absent in the plant enzyme.^71^ α-1,4-Glucan phosphorylases have been used to synthesise many different glucan-based molecules (Fig. 11),^72^ including: extension of maltoheptaose acceptor covalently immobilised on chitosan (**72**)^73^ or polystyrene (**73**);^74^ twining polysaccharides around a hydrophobic core to form a macromolecular complex, such as amylose-wrapped lipid;^75^ extension of amylopectin fragments immobilised on gold surfaces, giving rise to starch-like materials.^68^ These enzymes have allowed the production of hybrid materials, such as: novel HPLC matrices based on extension of maltoheptaose immobilised on silica (**74**);^76^ soluble single-walled carbon nano-tubes, insulated by wrapping with amylose helices.^77^ This class of phosphorylase displays some promiscuity towards the donor substrate. 2-Deoxy-maltooligosaccharides (**75**)^78^ have been synthesised from d-glucal in the presence of Pi, which can then be phosphorolysed to synthesise 2-deoxy-α-glucose-1-phosphate (Fig. 11). Deoxy- and deoxyfluoro-d-glucose could be transferred on to glycogen by this class of phosphorylase, but in very low yield (**77**–**79**).^79^ Alternative sugar-1-phosphates can be utilised as donor substrates, including those derived from d-xylose (**80**),^80^ d-mannose **(81**),^81^ d-glucosamine (**82**),^82^ *N*-formyl-d-glucosamine (**83**)^83^ and d-glucuronic acid (**84**).^84^ In each of these cases, the products isolated were all the result of a single residue extension, indicating that these sugar-extended products were not themselves good acceptor substrates of the enzyme for further extension.

### 7.2 Maltose phosphorylase

**Figure 12:**
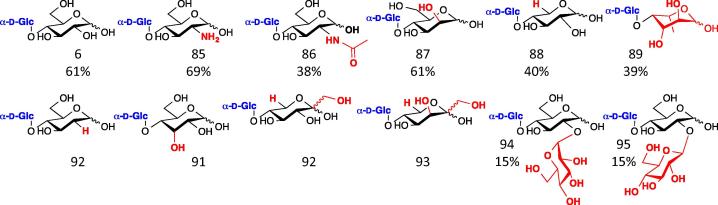
Products formed using maltose phosphorylase. Glucose was transferred onto (product numbers in brackets) the 4 position of d-Glc (**6**), d-glucosamine (**85**), d-GlcNAc (**86**), d-mannose (**87**), d-xylose (**88**) and l-fucose (**89**).^146^ 2-Deoxy-d-glucose (**90**), d-allose (**91**), d-sorbose (**92**) and d-tagatose (**93**) were also noted as acceptors, though the products were not characterised.^87^ Kojibiose (Glc-α-1,2-Glc) (**94**) and sophorose (Glc-β-1,2-Glc) (**95**) were also acceptors.^89^

Maltose phosphorylase was initially identified in *Neisseria meningitidis* as an enzyme that degrades maltose (**6**) to β-d-Glc-1-P and d-Glc.^85^ It was found to act exclusively on this disaccharide in a phosphorolysis sense^86^ but was reasonably promiscuous towards acceptor monosaccharide in glycosylation reactions (Fig. 12).^87^ The selectivity for the disaccharide formed during glycosylation reactions can be altered to give kojibiose (**2**) or trehalose (**1**) by the engineering of a specific loop which forms part of the enzyme active site.^88^

A maltose phosphorylase has been identified in *Bacillus selenitireducens* which is capable of using kojibiose and sophorose as acceptors, forming the expected trisaccharides (**94**–**95**) with isolated yields in the 15% range.^89^

### 7.3 α-1,4-Glucan:maltose-1-phosphate maltosyltransferase

An enzyme was identified in Mycobacteria which catalyses the transfer of maltose from α-maltose-1-P on to glycogen.^90,91^ This is the only established phosphorylase that operates in a glycoside synthesis sense in vivo and the only phosphorylase which catalyses the transfer of a disaccharide, iteratively extending the glucan chain by two residues at a time.^91^ This enzyme can also use maltosyl-fluoride as a donor. The structure of the enzyme shows a large and defined carbohydrate binding cleft, indicating limited substrate flexibility.^92^ Nonetheless, this enzyme unexpectedly forms fluorinated maltooligosaccharides when fed 2-deoxy-2-fluoro-maltosyl fluoride.^93^

## 8 β-1,4-d-Glycan phosphorylases

Cellulose represents an enormous renewable resource but it is difficult to process. Many microorganisms have developed sophisticated capabilities for degrading this recalcitrant material, including direct oxidation^94,95^ and multi-component cellulosomes.^96^ The cellodextrins released by cellulose hydrolases can be imported directly into cells,^97^ where they are further broken down, either by hydrolysis to glucose and cellobiose, or by cellobiose phosphorylase-mediated phosphorolysis to release d-Glc and α-d-Glc-1-P.^98,99^ β-1,4-Glycan disaccharide phosphorylases, which have been more thoroughly studied than the corresponding oligosaccharide phosphorylases, include phosphorylases acting on d-galactosyl l-rhamnose (**146**),^100^ d-mannosyl d-glucose (**152**),^101^ d-mannobiose (**160**),^102^ d-chitinbiose,^103^ d-cellobionic acid (**172**)^104^ and d-cellobiose (**7**).^99^

### 8.1 Cellobiose phosphorylase

**Figure 13:**
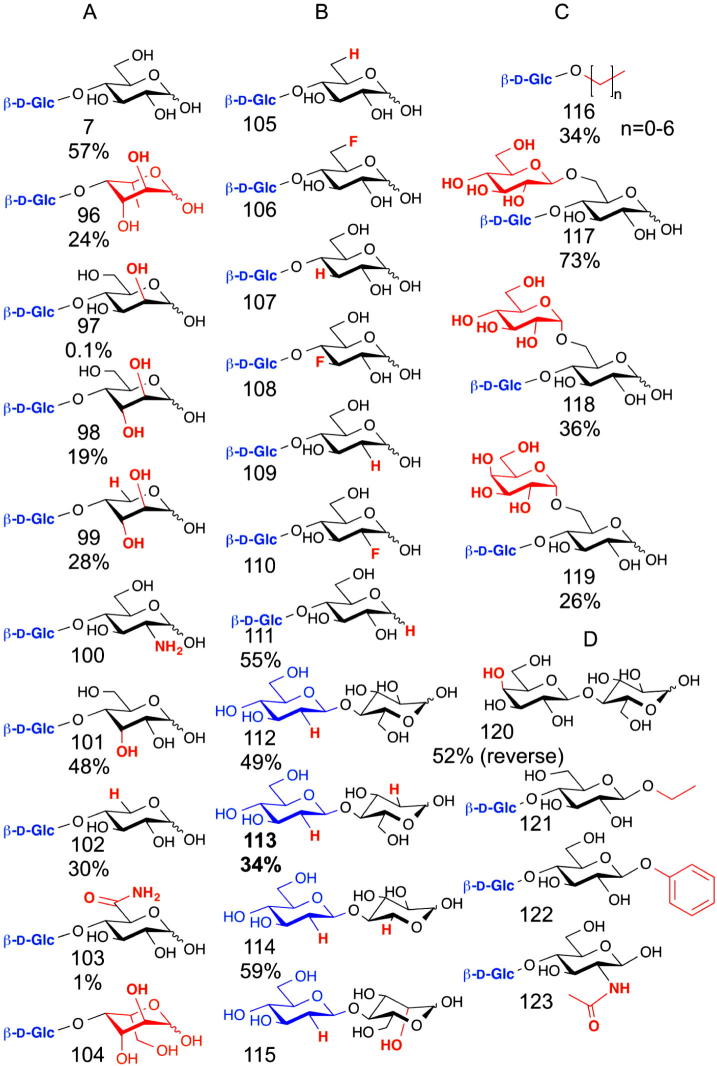
Proposed products formed using cellobiose phosphorylases. (A) Glc can be transferred (product numbers in brackets) from Glc-1-P on to various monosaccharides including glucose (**7**), l-fucose (**96**), d-allose (**97**), d-altrose (**98**),^106^d-arabinose (**99**),^110^d-glucosamine (**100**), d-mannose (**101**), d-xylose (**102**),^108^d-glucuronamide (**103**)^112^ and l-galactose (**104**).^105^ (B) Deoxy and fluoro-cellobioses could also be made using 6-deoxy (**105**), 6-fluoro- (**106**), 3-deoxy- (**107**), 3-fluoro- (**108**), 2-deoxy- (**109**) and 2-fluoro- (**110**) d-glucose as acceptors.^107^ 1-Deoxy-d-glucose, known as 1,5-anhydroglucitol, also accepted glucose to form 1,5-anhydrocellobiitol (**111**).^113^ When glucal was used as the donor 2′-deoxy-cellobiose (**112**), 2,2′dideoxy cellobiose (**11**3) and 2′deoxy-glucosyl d-xylose (**114**) and d-mannose (**115**) were formed using appropriate acceptors.^111^ (C) Alkyl chains (**116**) were acceptors^114^ as were the 6-glycosylated glucoses gentiobiose (**117**), isomaltose (**118**) and melibiose (**119**).^110^ (D) Mutagenesis of cellobiose phosphorylase generated lactose phosphorylase, which generated Gal-1-P from lactose (**120**),^115^ allows addition of substituents to the reducing end glucose, as in β-ethyl (**121**) or β-phenyl (**122**) glucosides,^116^ and allows the addition of bulky *N-*acetyl at the 2 position (**124**).^117^ Yields are indicated below for some of the products, but for some only kinetic parameters were reported. Gal-1-P was obtained from lactose (**120**) in 52% yield.

Of the β-1,4-d-glycan phosphorylases noted above, cellobiose phosphorylase (CBP) has been particularly heavily studied with respect to substrate specificity. Whilst many assays utilise only phosphate release to indicate turnover,^105^ purification of the products is required in order to confirm phosphorolysis product formation, rather than hydrolysis of the donor. 3-Deoxy-, 3-deoxy-3-fluoro- and the 3-epi-d-glucose (allose) are confirmed acceptors for CBP, giving **107**, **108** and **101**, respectively, but 3-*O*-methyl d-glucose is not, suggesting steric constraint around the 3OH group in the enzyme–substrate complex (Fig. 13).^106^ Detailed substrate interactions were studied with a range of deoxy- and fluoro-sugars, all showing reasonable turnover (forming **105**–**111)** except the 3-deoxy-3-fluoro-d-glucose, which was a poor acceptor.^107^ Glycoside products were also obtained from d-mannose (**97**), d-glucosamine (**100**) and d-xylose,^108^ which gave d-glucosyl-β-1,4-d-xylose (**102**) in a 60% yield using a cell extract of *Cellvibrio gilvus*.^109^ A 28% yield of disaccharide (**99**) (Fig. 13) was achieved from the transfer of Glc from Glc-1-P on to d-arabinose.^110^ d-Glucal was also a substrate for cellobiose phosphorylase in the presence of Pi, allowing formation of 2′-deoxyglucosyl disaccharides (**112**–**115**).^111^ In addition, trisaccharides could be synthesised from 1,6-disaccharides, giving β-1,4-linked glycosides from gentiobiose (**117**), melibiose (**119**) and isomaltose (**118**) as acceptors for glucosyl transfer.^110^ Further, whilst d-glucuronic acid was not an acceptor for cellobiose phosphorylase, d-glucuronamide was successfully transformed into the corresponding disaccharide (**103**), although in very low yield (∼1%).^112^ 1,5-Anhydro-glucitol^113^ and linear alcohols as small as methanol and as large as heptanol were utilised as acceptors, giving (**111**) and (**116**), although the branched alcohols isopropanol and *tert*-butanol were not substrates.^114^

Mutagenesis of the cellobiose phosphorylase donor binding site (T508I/N667A) allowed the formation of a lactose phosphorylase, capable of synthesising *α*-glactose-1-phosphate from lactose (**120**) in the presence of inorganic phosphate.^115^ Another mutant, with additional alterations in the acceptor binding site and the active site entrance (N156D/N163D/E649G), allowed the transfer of glucose onto anomerically substituted glucosides,^116^ whereas the removal of a bulky tyrosine residue allowed an *N*-acetyl group at the two position of the acceptor glucose, facilitating synthesis of d-Glc-β-1,4-d-GlcNAc (**123**).^117^

### 8.2 Cellodextrin phosphorylases

**Figure 14:**
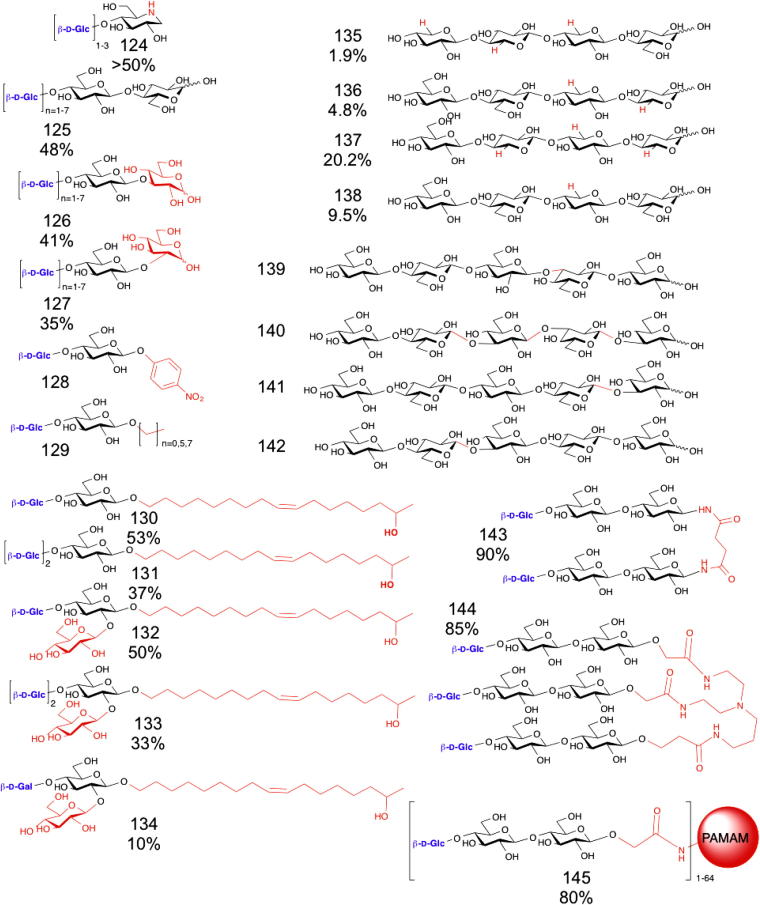
Proposed products formed using cellodextrin phosphorylase. Cellodextrin phosphorylase can be used to extend (product numbers in brackets) deoxynojirimycin (DNJ) to form pseudo cellooligosaccharides for use as cellulase inhibitors (**124**), with a combined yield of >50%.^120^ Trisaccharides could be purified from the transfer of glucose on to the 4 hydroxyl of cellobiose (**125**), sophorose (**126**) and laminaribiose (**127**).^110^ The enzymes were also capable of extending glucosides including *p*-nitrophenol glucoside (**128**) and β-alkyl glucosides (**129**).^122^ Novel glycolipids could be made by adding glucose to glucosyl (**130**–**131**) and sophorsyl (**132**–**133**) lipids and a single transfer from galactose-1-P on to the glucosyl lipid was achieved, yielding lactosyl lipid (**134**).^123^ Using either d-Xyl-1-P or d-Glc-1-P as the sugar donors and the synthesised xylo- and cellooligosaccharides as acceptors specific xyloglucan oligosaccharides can be synthesised (**135**–**138**). ^121^ β-1,3/1,4 Glucans can be made using laminarin phosphorylase and CDP (**139**–**142**).^55^ Dendrimers were also made by extending cellobiose derivatised succindiamide (**143**), tris(2-aminoethyl)amine (**144**) and polyamidoamine (**145**) with CDP, though the products were not isolated.^124^ For products that were purified yields are indicated.

The most studied polymerising β-1,4-glucan phosphorylase is cellodextrin phosphorylase (CDP),^118^ which is closely related (41% similarity) to the cellobiose phosphorylases. This enzyme has been used to synthesise crystalline cellulose^119^ and cellooligosaccharide derivatives of the iminosugar glycosidase inhibitor deoxynojirimcin (DNJ, giving **124**) for assessment as cellulase inhibitors (Fig. 14).^120^ A range of β-linked disaccharides acted as acceptor substrates for CDP (see **126** and **127**, for instance),^110^ showing that the enzyme is permissive of anomeric substitution in β-d-glucosyl acceptors.

In addition to using d-Glc-1-P as a donor, CDP was also capable of transferring d-xylose from d-Xyl-1-P, and could also transfer either d-Glc or d-Xyl on to β-linked d-xylose, facilitating the synthesis of a series of xylans related to hemicellulose (Fig. 14 and **135**–**138**).^121^ By alternating between CDP and laminarin phosphorylase (vide supra), both of which will transfer on to many β-glucans, the synthesis of a set of defined β-1,3/1,4-glucans related to plant cell walls was achieved (**139**–**142**).^55^ PNP (**128**) and alkyl **(129**) celloglycosides were also assessed as acceptors for CDP,^122^ which also proved capable of synthesising novel glycolipids (**130**–**132**).^123^ Dendrimers based on cellulose could readily be synthesised by the extension of cellobiose dendrimer core structures (**143**–**145**).^124^

### 8.3 d-Galactosyl l-rhamnose phosphorylase

**Figure 15:**
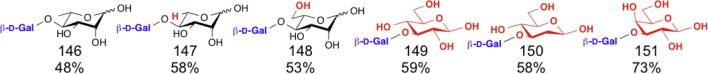
Disaccharides formed using galactosyl rhamnose phosphorylase. Galactosyl rhamnose phosphorylase adds glucose (product numbers in brackets) to the 4 hydroxyl of l-rhamnose (**146**), l-lyxose (**147**) and l-mannose (**148**) and to the 3 position of d-glucose (**149**), 2-deoxy d-glucose (**150**) and d-galactose (**151**).^100^ Xylose is also noted as an acceptor but the linkage of the product is not defined.^147^

An enzyme has been identified which transfers d-galactose on to l-rhamnose more efficiently than onto any other sugar, leading to its proposed identification as a β-1,4-d-galactosyl l-rhamnose phosphorylase.^100^ This galactosyl rhamnose (**146**) structure is found in the cell walls of plants, such as *Tanacetum vulgare*,^125^ and bacteria, including *Klebsiella* strains,^126^ and these may represent the natural substrates for this enzyme. In vitro this enzyme was found to preferentially transfer d-galactose from d-Gal-1-P onto the 4-OH of l-*rhamno*-configured sugars with relaxed specificity at the 6-position (see **146**–**148**).^100^ It also transferred onto the 3-OH of d-sugars with flexibility around the substituents at the 2 and 4 positions (see **149**–**151**) (Fig. 15).

### 8.4 β-1,4-d-Mannan phosphorylases

Plant cell walls contain large amounts of hemicellulose, including β-1,4-mannan. This material is structurally related to cellulose and can be hydrolysed to mannodextrins and mannobiose, which can be directly phosphorolysed by mannan phosphorylase or mannobiose phosphorylase, respectively.^102^ Mannobiose can also be epimerised by cellobiose 2-epimerase to mannosyl glucose (**152**), which can subsequently be phosphorolysed by mannosyl-glucose phosphorylase, releasing α-d-Man-1-P and d-Glc.^101^

**Figure 16:**
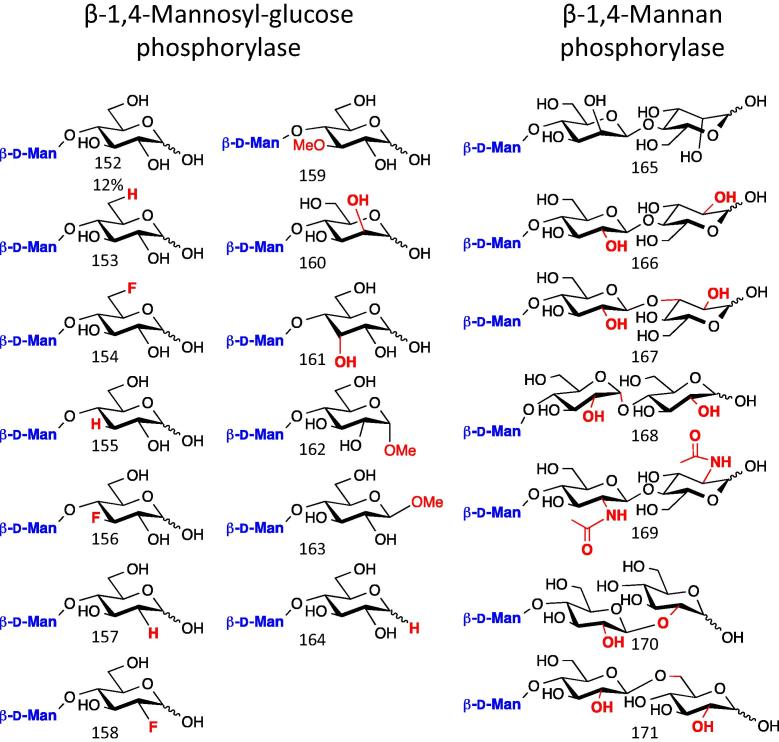
Prospective products formed using mannan phosphorylases. Mannosyl-glucose phosphorylases have been used to add (products numbers in brackets) d-mannose β-1,4 to d-glucose (**152**), 2-, 3- and 6- deoxy and deoxyfluoro-d-glucose (**153**–**158**), 3-*O*-methyl d-Glc (**159**), d-Man (**160**), d-allose (**161**), d-glucose α and β-methyl glycosides (**162**–**163**) and 1,5-anhydro d-glucitol (**164**).^102^ Mannan phosphorylase transferred at least one mannosyl unit onto mannobiose (**165**), cellobiose (**166**), laminaribiose (**167**), maltose (**168**), chitinbiose (**169**), gentiobiose (**170**) and sophorose (**171**).^102^ Isolated yields are indicated below for some of the products, but for many reactions only kinetic parameters were reported.

Mannan phosphorylases have been used to synthesise a limited number of β-1,4-d-Man-terminating disaccharides, consistent with their promiscuity towards modification at the C-1, C-2, C-3 or C-6 positions of the acceptor (Fig. 16).^102^ *Ruminococcus albus* produces a mannosyl glucose phsophorylase which shows some promiscuity towards the 6 position of the acceptor glucose moiety.^102^ Recently a structure of the *Bacteroides fragilis* mannosyl glucose phosphorylase was solved, leading to the proposal of a novel proton shuttle catalytic mechanism in which the mannose 3OH protonates the leaving group, restricting the potential to modify this position of prospective substrates.^127^

### 8.5 Chitinbiose phosphorylase

*Vibrio* species degrade the d-GlcNAc-β-1,4-d-GlcNAc linkage found in chitin by secreting hydrolases that form GlcNAc and chitinbiose, where the former is then imported into the cell^128^ and the latter is degraded by hydrolysis or by phosphorolysis to release d-GlcNAc-1-P.^103^ The *N*,*N*′-diacetylchitobiose phosphorylase involved in this process has been found to transfer d-GlcNAc from d-GlcNAc-1-P onto d-GlcNAc and *p*-nitrophenyl and methylumbelliferyl β-glycosides of d-GlcNAc.

### 8.6 d-Mannosyl (*N*-acetyl-d-glucosamine) phosphorylase

Recently a novel phosphorylase was found in the human gut microbe *Bacteroides thetaiotaomicron* that is able to degrade complex human *N*-glycans.^129^ This phosphorylase is capable of phosphorolysis of the d-Man-β-1,4-d-GlcNAc released by hydrolysis of *N*-linked glycans. This core linkage is difficult to synthesise and thus this enzyme may prove valuable in the synthesis of *N*-glycan core structures, particularly as it is able to transfer Man onto GlcNAc-β-1,4-GlcNAc.^129^

### 8.7 Cellobionic acid phosphorylase

**Figure 17:**
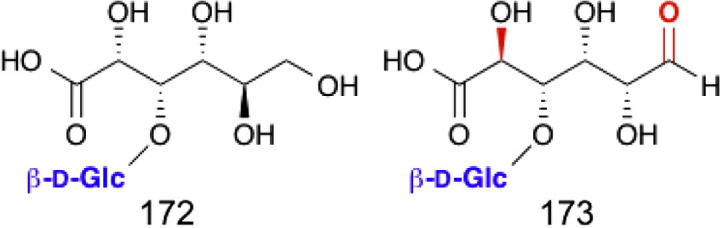
Products of cellobionic acid phosphorylase. Cellobionic acid phosphorylase can transfer glucose onto the 4 position of gluconic acid (giving **172**) or the 3 position of glucuronic acid (giving **173**), which is in the same position with respect to the carboxylic acid moiety.

Recently novel enzymes identified in the fungus *Neurospora crassa* and the bacterium *Xanthomonas campestris* were shown to phosphorolyse the d-glucopyranosyl-β-1,4-d-gluconic acid (cellobionic acid)^104^ liberated by cellulose lyases. These enzymes were only capable of transferring d-glucose on to the 4 position of gluconic acid (giving **172**) or the 3 position of glucuronic acid (giving **173**), which is explained by binding of the carboxylate in the same orientation in the enzyme (Fig. 17).

## 9 Future directions

Phosphorylases are highly versatile enzymes, which can readily be used to synthesise both simple glycosides and large, complex oligosaccharide chains. However, there are only a limited number of these enzymes currently known and re-engineering them to access novel glycan structures, whilst attractive, has limitations, with lower activity and often lower regiospecificity than wild type being observed to date.^130^ The likely diversity of activities already present in nature remains an attractive but untapped resource.

### 9.1 Identification of the missing glucan phosphorylases

There are no known phosphorylases for β,β-trehalose, but this linkage has not so far been found in Nature and thus it might not be possible to find a β-1,1-phosphorylase. On the other hand, α-1,6 and β-1,6-glucans are rather common in Nature, produced by bacteria in dental plaque^131^ and in the yeast cell wall,^132^ for example, although no corresponding phosphorylases have yet been discovered. It seems reasonable to expect that phosphorylases may exist for these linkages and could be found by assaying organisms that can use linear dextrans as a sole carbon source, for instance.

### 9.2 Identification of phosphorylases for non-glucosides

Most of the phosphorylases so far identified act on d-glucosides. This class of carbohydrates makes up the major energy storage and structural polysaccharides used in Nature but there are numerous other major naturally occurring polysaccharides: fructans are storage polymers of fructose found in some plants and algae;^133^ plant cell walls contain many complex polymers, including xylans and uronic acid polymers;^134^ animals cells are covered in layers of charged polysaccharides, such as chondroitin and heparin;^135^ many types of seaweed have complex and charged polymers in their cell wall.^136^ With the large amounts of these compounds produced globally, it is likely there are many phosphorylases yet to be identified that act on these materials. In order to find them, microbes that can use these alternative polysaccharides as carbon sources could be screened. Since many of these compounds are useful in biotechnology, food and medicine the possibility of synthesising defined components, as offered by phosphorylases, is highly attractive.

### 9.3 Engineering disaccharide phosphorylases for glycopolymer synthesis

Many of the phosphorylases so far studied act on disaccharides. This limits their use in polysaccharide biotechnology and the possibility of converting them into polymerising phosphorylases is highly attractive. It is likely that disaccharide phosphorylases will continue to yield to biochemical analysis more readily than their polymerising counterparts. The ability to convert them into polymerising enzymes therefore represents an attractive prospect for polysaccharide synthesis. Mutagenesis studies have been used to enhance the flexibility of disaccharide phosphorylase enzymes: opening up the active site of CBP to allow glycosides of glucose to act as acceptor substrates^116^ and increasing the length of koji-oligosaccharides produced by kojibiose phosphorylase^28^ have both been reported.

Projecting ahead, comparison of phosphorylases from the same CAZy family but with different acceptor lengths may inform mutations and the generation of chimeras to produce novel polymerising phosphorylase enzymes. Cellobiose phosphorylase and cellodextrin phosphorylase are both in the same CAZy family, GH94, so comparison between their structures may help to define the features that determine substrate length. In a similar sense, chitinbiose phosphorylase could be altered by the generation of chimeric proteins with CDP; a nigerose phosphorylase may be compatible with the generation of polymerising kojibiose phosphorylase chimeras, since they are in the same GH family. The lessons learnt from such studies might provide design guidelines that may be extended to those families of phosphorylase that do not contain a polymerising activity.

### 9.4 Relationship between hydrolases, transferases and phosphorylases

Most of the characterised glycan phosphorylases are members of CAZy glycosyl hydrolase families,^7^ although it is unclear whether these phosphorylases evolved from hydrolases or vice versa. Logic dictates there should be a hydrolase (or phosphorylase) for every sugar linkage in nature, barring any that are exclusively degraded by lyases. There are many more hydrolases characterised than phosphorylases. If typically robust hydrolases could be engineered into phosphorylases, then the repertoire of glycosidic linkages that could be readily synthesised enzymatically from simple sugar phosphates would be enormous.

**Figure 18:**
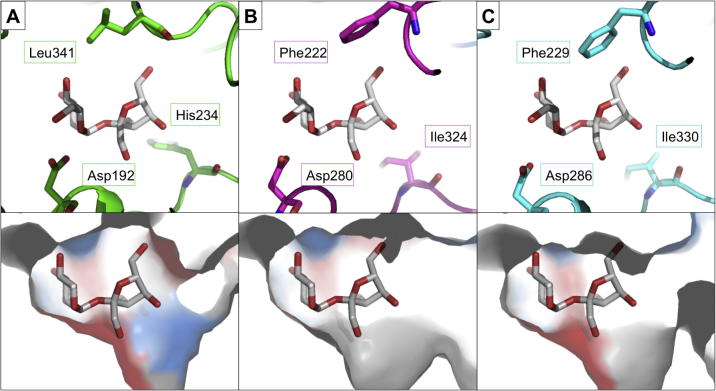
Family GH13 active site. Sucrose phosphorylase (green, 2GDV)^148^ has a similar active site to sucrose hydrolase (magenta, 3CZK)^149^ and amyloglucosidase (cyan, 1G5A).^150^ However one loop has moved and there are two important amino acid changes—Leu341 and His234, which gives a positively charged patch in the active site. Sucrose modelled from 3CZK. (For interpretation of the references to colour in this figure legend, the reader is referred to the web version of this article.)

In order to understand the similarities and differences between phosphorylases and hydrolases, detailed structural analysis of closely related enzymes is required. Amylosucrase, sucrose hydrolase and sucrose phosphorylase are all members of the GH13 family, for instance. The phosphorylase structure shows a slight deviation in one active site loop and there are notably His234 and Leu341 in the active site, not Ile and Phe, as in the hydrolase and *trans*-glycosylase structures (Fig. 18).^137^

As there is no phosphate bound in the phosphorylase structure, it is not possible to say with confidence what the impact of these alterations is, but it is likely that the histidine is important for the coordination of the phosphate in the active site. Further crystallographic analysis is required to understand phosphate localisation and exclusion of H_2_O. In principle, mutagenesis and chimera generation studies could be used to effect the interconversion of hydrolases and phosphorylases. There are α-1,6-hydrolases in the family GH13 and it might be possible to convert these enzymes into phosphorylases, based on the strategies alluded to above. There are, however, currently no characterised enzymes in this family that act on substrates other than glucosides.

Trehalose phosphorylase and trehalose hydrolase are both members of the GH65 family. The structure of the hydrolase is not yet known; its comparison to the hydrolase structure may highlight important structural features in this family. There are no other reported hydrolases in this family, however, and so there is only limited application of the principles elucidated for the development of phosphorylases in this family.

Beyond GH13 and GH65, there are currently no other hydrolase families that also contain phosphorylases, so the rules developed from families GH13 and 65 would need to then be tested more widely. It is likely that the exclusion of water is the most important aspect when engineering these enzymes and the development of glycosynthases provides useful insight and direction for this work.^138^

In addition to comparison of glycoside hydrolases and phosphorylases, one needs to take into account that the polymerising α-1,4-glucan phosphorylases are actually found in a glycosyltransferase family (GT35). As GT35 contains exclusively phosphorylases, there is no clear framework for the development of novel activities based on these structures. However in the GT4 family, in addition to trehalose phosphorylase a wide range of α-glycosyl transferases are present. Information on the specific features which control transfer of sugars from nucleotides or phosphate may inform the development of novel phosphorylases in this family.

## 10 Conclusion

Phosphorylases represent a wide range of highly flexible enzymes that can be used to synthesise, or phosphorolyse, a diverse range of carbohydrate structures. Significantly, this class of enzyme has already been utilised for the commercial synthesis of 2-*O*-α-d-glucosyl glycerol^4^ and for the kilogram scale preparation of lacto-*N*-biose.^5^ With the increasingly high speed and low cost of modern genome sequencing technologies, more of these enzymes are being discovered, which will add substantially to the toolbox of carbohydrate-active enzymes available for synthesis applications.

## Figures and Tables

**Table 1:** Classification of characterised glycan phosphorylases. Type R = retaining, I = Inverting. NA = has not been defined.

Linkage	Name	CAZy family	Type	Chain length for phosphorolysis	EC	Refs.
α,α-1,1	Trehalose (d-Glc-d-Glc)	GH65	I	2	2.4.1.64	16
α,α-1,1	Trehalose-6-P (d-Glc-d-Glc6P)	GH65	I	2	2.4.1.216	17
α,α-1,1	Trehalose (d-Glc-d-Glc)	GT4	R	2	2.4.1.231	19
α-1,2	Kojibiose (d-Glc-d-Glc)	GH65	I	2+	2.4.1.230	25
α,α-1,2	Sucrose (d-Glc-d-Fru)	GH13	R	2	2.4.1.7	31
α-1,2	Glucosyl glycerol (d-Glc-d-glycerol)	GH65	I	2	2.4.1.-	38
β-1,2	β-1,2-Glucan (d-Glc-d-Glc)	GH94	I	NA	2.4.1.-	43
α-1,3	Nigerose (d-Glc-d-Glc)	GH65	I	2	2.4.1.279	44
α-1,3	Glucosyl rhamnose (d-Glc-l-Rha)	GH65	I	2	2.4.1.282	46
β-1,3	Laminaribiose (d-Glc-d-Glc)	GH94	I	2	2.4.1.31	49, 56
β-1,3	Laminarin (d-Glc-d-Glc)	NA	I	2+	2.4.1.30	51
β-1,3	β-1,3-Glucan (d-Glc-d-Glc)	NA	I	2+	2.4.1.97	52, 53
β-1,3	Galactosyl HexNAc (d-Glc-d-GlcNAc/d-GalNAc)	GH112	I	2	2.4.1.211	60, 62
α-1,4	Glycogen/ starch (d-Glc-d-Glc)	GT35	R	4+	2.4.1.1	139, 140
α-1,4	Maltose (d-Glc-d-Glc)	GH65	I	2	2.4.1.8	85
α-1,4	α-1,4-Glucan:maltose-1-P maltosyltransferase ((d-Glc)_2_-d-Glc)	GH13	R	6+	2.4.99.16	90
β-1,4	Cellobiose (d-Glc-d-Glc)	GH94	I	2	2.4.1.20	99
β-1,4	Cellodextrin (d-Glc-d-Glc)	GH94	I	2+	2.4.1.49	98
β-1,4	Galactosyl rhamnose (d-Gal-l-Rha)	GH112	I	2	2.4.1.-	100
β-1,4	Mannosyl glucose (d-Man-d-Glc)	GH130	I	2	2.4.1.281	101
β-1,4	Mannan (d-Man-d-Man)	GH130	I	2+	2.4.1.-	102
β-1,4	Chitinbiose (d-GlcNAc-d-GlcNAc)	GH94	I	2	2.4.1.280	103
β-1,4	Mannosyl GlcNAc (d-Man-d-GlcNAc)	GH130	I	2	2.4.1.-	129
β-1,4	Cellobionic acid (d-Glc-d-GlcA)	GH94	I	2	2.4.1.-	104
